# The Lipid Accumulation Product Index (LAP) and the Cardiometabolic Index (CMI) Are Useful for Predicting the Presence and Severity of Metabolic Syndrome in Adult Patients with Obesity

**DOI:** 10.3390/jcm13102843

**Published:** 2024-05-11

**Authors:** Sofia Tamini, Adele Bondesan, Diana Caroli, Alessandro Sartorio

**Affiliations:** 1 Istituto Auxologico Italiano, Istituto di Ricovero e Cura a Carattere Scientifico (IRCCS), Experimental Laboratory for Auxo-Endocrinological Research, 28824 Piancavallo-Verbania, Italy; 2 Istituto Auxologico Italiano, Istituto di Ricovero e Cura a Carattere Scientifico (IRCCS), Experimental Laboratory for Auxo-Endocrinological Research, 20145 Milan, Italy

**Keywords:** obesity, metabolic syndrome, lipid accumulation product index, cardiometabolic index, body adiposity index

## Abstract

**Background**: The concomitant occurrence of obesity and metabolic syndrome (MetS) causes a significant worsening of a patient’s clinical condition. Indexes that employ anthropometric measurements alone or associated with blood parameters have been investigated for their ability to identify MetS. This study aimed to evaluate the diagnostic accuracy of three of these indexes, the body adiposity index (BAI), the lipid accumulation product index (LAP), and the cardiometabolic index (CMI), in a cohort of 1912 adult subjects with obesity. **Methods and Results**: MetS was found in 62.3% of the enrolled subjects, with a higher prevalence in males (72.5%) than females (60.9%). Receiver operating characteristic (ROC) analysis was used to define which index performed better. The BAI was found to be the lowest-performing index, with an ROC area of 0.50, a sensitivity of 30.31%, a specificity of 74.48%, and a likelihood ratio of 1.19. On the contrary, the LAP and the CMI showed a comparable ROC area of 0.82. The LAP had a sensitivity of 63.06%, a specificity of 86.55%, and a likelihood ratio of 4.69, while the CMI had a sensitivity of 67.59%, specificity of 81.55%, and a likelihood ratio of 3.66. The analysis was also performed in the group divided into males and females, with overlapping results. **Conclusions**: The LAP and the CMI performed better than the BAI in detecting MetS both in the general population with obesity and in the male/female subgroups. In the future, it will be important to validate these useful diagnostic tools in order to employ them in clinical practices.

## 1. Introduction

Obesity is a chronic disease of pandemic proportions, complex and multifactorial, and its prevalence and severity have significantly increased in recent decades, and have undergone further growth and worsening following the COVID-19 pandemic [1,2]. Obesity, particularly visceral obesity, is strongly associated with the onset of numerous comorbidities [3]. In fact, this condition adversely affects nearly all physiological functions of the body, leading to an increase in the risk of developing multiple other conditions, such as type 2 diabetes mellitus (T2DM), cardiovascular diseases, various types of cancers, musculoskeletal disorders, dyslipidemia, non-alcoholic fatty liver disease, and metabolic syndrome (MetS) [4,5]. The majority of these complications are represented by metabolic disorders, which are characterized by a state of chronic inflammation [6,7] and are closely interconnected. These complications determine the worsening of the patient’s clinical condition and possibly result in the development of serious cardiovascular complications [8]. Furthermore, the presence of both obesity and MetS can induce atherosclerosis, with the consequence of a significant increase in the risk of cardiovascular mortality [9,10,11,12]. According to different epidemiological studies, the prevalence of MetS in the obese population is usually approx. 35%, and the incidence of it is expected to increase rapidly. Moreover, the risk for it is higher in women (34.4%) compared to men (29%) and increases with age. On the other hand, the prevalence of MetS in individuals with a normal weight is approx. 5% [13,14,15].

For this reason, it is necessary to find suitable tools for the identification of those subjects with obesity who are at a higher risk of developing such metabolic alterations, particularly MetS. The simplest and fastest screening method is the use of predictive indexes, which do not require complex and invasive examinations or tests but are capable of discriminating the presence or absence of risk factors with sufficient precision and sensitivity.

Over the years, various indexes have been developed for this purpose, the first being the body mass index (BMI), which is the most used index in clinical practice [16]. Nonetheless, the BMI has many different limitations since it does not discriminate between fat mass and fat-free mass, or fat distribution [17,18,19], which are considered to be the most important predictors of cardiometabolic risk. Due to these limits, alternative indexes have been proposed, which comprehend both anthropometric features alone and in combination with lipid measurements, specifically the body adiposity index (BAI) [20], the lipid accumulation product index (LAP) [21], and the cardiometabolic index (CMI) [22]. 

The BAI was proposed as an anthropometric tool to evaluate adiposity to overcome a weakness of the BMI and can be calculated solely from hip circumference and height. It can also be used to reflect body fat percentages in adults [20]. The LAP is an index developed to assess the accumulation of lipids, especially the central accumulation of them, with the ability to predict the degree of lipotoxicity. It is calculated using waist circumference, as an indicator of visceral adiposity, and fasting circulating triglyceride levels, which are the esterified, long-chain fatty acids that circulate through the blood and are contained stably inside lipoproteins [21]. Both these indexes have been suggested as early markers of metabolic dysfunction and seem to have greater clinical utility than the BMI in predicting metabolic disorders, including MetS and T2DM, in both individuals of different ethnicities who have obesity or have a normal weight [21,23,24,25,26,27,28,29]. The LAP is considered to be a clinically useful marker for the estimation of insulin resistance and cardiometabolic risk, being also associated with NAFLD and arterial stiffness. Moreover, it also showed high accuracy in the diagnosis of MetS in healthy individuals [21,30,31,32,33,34,35].

In addition to the more studied BAI and LAP, the CMI has been developed as another and more complex indicator since it takes into account both adiposity and blood lipids. In particular, it is calculated as the product of the waist/hip ratio and the triglyceride/high-density lipoprotein ratio. This index considers both the accumulation of abdominal fat and the presence of dyslipidemia in order to identify a potential risk of metabolic alterations, including the presence of MetS and/or T2DM [22]. Previous studies found that CMI values were closely associated with cardiovascular and kidney diseases, being also considered as a promising predictive parameter of metabolism-related diseases [36,37,38,39,40].

The necessity of adopting inexpensive methods for directly measuring fat mass and visceral adiposity is the main reason for using these surrogate indexes. However, there is no consensus regarding which of these indexes exhibit a better performance, specifically in identifying MetS in the adult population with obesity.

For these reasons, the present study aimed to evaluate the ability and compare the accuracy of these three indexes (the BAI, the LAP, and the CMI) to identify the presence of MetS in a large cohort of Caucasian adult patients with obesity seeking an in-hospital multidisciplinary body-weight reduction program.

## 2. Materials and Methods

### 2.1. Study Population

One thousand nine hundred and twenty-three Caucasian adults with obesity, hospitalized at the Division of Metabolic Diseases, Istituto Auxologico Italiano, IRCCS, Piancavallo (VB) for a 3 week multidisciplinary body-weight reduction program (BWRP) between January 2017 and June 2019, were admitted to the study. The inclusion criteria were i. individuals of both sexes aged ≥ 18 years; ii. a BMI > 35 kg/m^2^; iii. the presence of the complete information needed for the calculation of the indices taken into consideration. All subjects had a full medical history and a physical examination, as well as routine hematology and biochemistry screenings and urine analysis. All subjects suffered from essential obesity, other genetic, organic, endocrine, or iatrogenic forms having been excluded. None of the subjects had taken weight-loss drugs during the 12 months before their enrollment into the present study.

**Figure 1:**
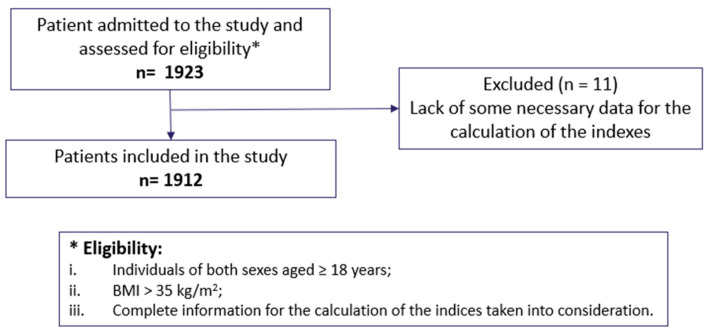
Consort flow diagram and eligibility criteria.

Eleven subjects were excluded from the study population due to the lack of some necessary data for the calculation of the indexes taken into consideration, thus making the final study population of 1912 individuals, as shown in Figure 1.

The study was approved by the Ethical Committee of Istituto Auxologico Italiano, Milan, Italy (ethical identification code: 2023_03_21_04, research code: 01C315, acronym: INDEMETSOB). All patients had signed a written informed consent form for the anonymous use of all their clinical, anthropometric, and biochemical parameters for scientific purposes at their admission to our hospital. 

### 2.2. Anthropometry

Body weight (BW) and height were measured at the admission to the hospital following international guidelines [41] using a scale with a stadiometer (Wunder Sa.Bi., WU150, Trezzo sull’Adda, Italy) with subjects only wearing underclothes. The BMI was calculated as weight (kg)/height (m)^2^.

Waist circumference (WC) was measured at the midpoint between the last rib and the iliac crest and hip circumference (HC) was measured at the largest parts around the buttocks using a flexible tape measure.

### 2.3. Laboratory and Clinical Measurements

Blood samples (about 10 mL) were collected on the second day of hospitalization before the start of the BWRP (to avoid possible influences related to the BWRP itself), early in the morning after an overnight fast in standard tubes for serum. Levels of glucose, total (T-C), HDL (HDL-C), cholesterol, and triglycerides (Try) were measured by the same internal laboratory using standard methods (Roche Diagnostics, Monza, Italy). 

Systolic (SBP) and diastolic blood pressure (DBP) were measured twice (with 3 min intervals in-between) on the dominant arm with an aneroid sphygmomanometer (TemaCertus, Milan, Italy), by using appropriated sized cuffs for adult subjects with obesity. The mean values were calculated and rounded to the nearest 5 mmHg value.

According to the IDF criteria for the diagnosis of metabolic syndrome [42], adult patients with obesity were identified as having metabolic syndrome if they had three or more of the following altered factors:

i. Abdominal obesity (WC ≥ 102 cm for males; ≥88 cm for females);
ii. Elevated triglycerides: ≥150 mg/dL (1.7 mmol/L) or specific treatment for this
iii. lipid abnormality;
iv. Reduced HDL-C: <40 mg/dL (1.0 mmol/L) in males; <50 mg/dL (1.3 mmol/L) in females, or specific treatment for this lipid abnormality;
v. Increased BP: SBP ≥ 130 mmHg or DBP ≥ 85 mmHg and/or the treatment of previously diagnosed hypertension;
vi. An increased fasting plasma glucose (FPG) concentration ≥100 mg/dL (5.6 mmol/L) or previously diagnosed type 2 diabetes mellitus.

### 2.4. Indexes

The following indexes were calculated according to the respective formulas:

- BAI [20] (males/females) = (HC (cm)/height (m)^1.5^) − 18
- LAP [21] (males) = WC (cm) − 65 × Try (mmol/L)
-     (females) = WC (cm) − 58 × Try (mmol/L)
- CMI [22] (males/females) = (Try (mmol/L)/HDL-C (mmol/L))/(WC (cm)/HC (cm))

### 2.5. Statistical Analysis

Continuous variables were expressed as mean ± standard deviation and categorical variables as absolute and relative frequencies. The Shapiro–Wilk was performed to verify that all parameters were normally distributed.

Receiver operating characteristic (ROC) curves were generated to obtain the values of the area under the curve (AUC), with sensitivity, specificity, and 95% CI, for each index as a predictor of MetS to define their discriminatory accuracy. In order to identify the optimal cutoff, the Youden index [43] was calculated. The analysis was performed on the whole group and in the population stratified by gender.

The whole study group was divided into two subgroups based on the presence or absence of MetS (MetS+ and MetS−, respectively) and into males and females. All parameters were compared between the MetS+ and MetS− subgroups and between the male and female subgroups by using a *t*-Student test for unpaired data or Fisher’s exact test. 

Furthermore, to verify the correlation between the three indexes and the parameters and characteristics evaluated in the study population, Pearson’s correlation was performed, and the r and r squared (r^2^) values were calculated to identify the relationship between each index and the variables considered. An r value below ± 0.10 indicated a negligible correlation, a value between ±0.11 and ±0.39 indicated a weak correlation, a value between ±0.40 and ±0.69 indicated a moderate correlation, a value between ±0.70 and ±0.89 indicated a strong correlation, and a value above ±0.90 indicated a very strong correlation [44].

A level of significance of *p* < 0.05 was used for all data analysis. The statistical analysis was performed with Prism 10 GraphPad (GraphPad Software, Boston, MA, USA) and IBM SPSS (Version 29.0.1.0) (IBM, Armonk, NY, USA).

## 3. Results

A total of 1912 adults with obesity participated in the study, 1690 females and 222 males, with a mean age of 50.7 ± 14.1 years (age range: 18–83 years) and a mean BMI of 43.3 ± 6.2 kg/m^2^.

According to the IDF criteria, MetS was present in 1191 patients (62.3%). All subjects fulfilled the criteria for having central obesity, while high BP was present in 1405 subjects (73.5%), increased triglycerides values in 682 (35.7%), reduced HDL-C levels in 1043 (54.6%), and hyperglycemia in 658 (34.4%) patients. 

MetS was more frequent in older patients (*p* < 0.0001). In fact, when the population was divided into younger (i.e., age ≤ 50 years) and older (i.e., age > 50 years) groups, MetS was present in 51.1% and 71.4%, respectively.

Based on the presence/absence of MetS, the population was divided into two subgroups (MetS + and MetS−, respectively). The main characteristics of the study population and the two subgroups are shown in Table 1.

As expected, almost all parameters were significantly worse in the MetS+ subgroup than in the MetS− subgroup. In fact, the two subgroups were comparable in terms of hip circumference (HC), height, and BAI index values, while the MetS+ subgroup was significantly heavier (both in terms of body weight (BW) (*p* < 0.001) and BMI values (*p* < 0.0001) and older, with a greater waist circumference (WC), and higher values of glycemia, cholesterolemia, triglyceridemia and systolic and diastolic blood pressure (*p* < 0.0001). The LAP and the CMI were also higher in the MetS+ subgroup than in the MetS− subgroup (*p* < 0.0001), while HDL cholesterol levels were significantly lower in the MetS+ subgroup (*p* < 0.0001). 

The population was also divided into males and females. The characteristics of the male and female subgroups are shown in Table 2.

No significant differences were found in terms of the BMI, glycemia, and cholesterolemia values between the two genders. By contrast, males showed significantly higher WC, BW, height, triglycerides, the LAP, and CMI values (*p* < 0.0001), systolic (*p* < 0.001) and diastolic (*p* < 0.05) blood pressure and MetS frequency (*p* < 0.001) compared to females, while females were older (*p* < 0.001), with greater HC (*p* < 0.05), and with higher HDL-C and BAI values (*p* < 0.0001) than males.

**Figure 2:**
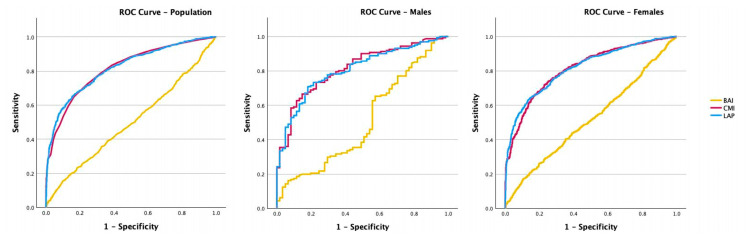
The ROC curve and the AUC comparing the ability of the BAI, the LAP, and the CMI to predict MetS in the whole population, in males, and in females.

**Figure 3:**
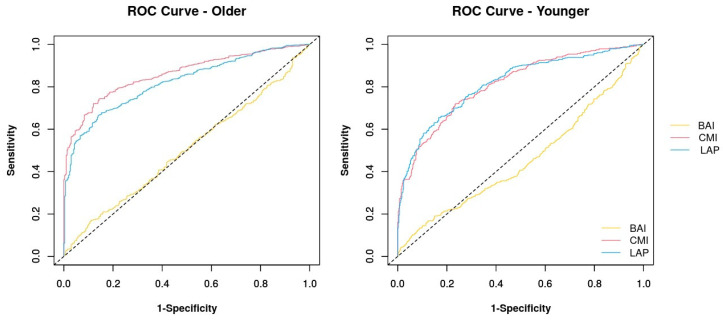
The ROC curve and the AUC comparing the ability of the BAI, the LAP, and the CMI to predict MetS in the younger (age ≤ 50 years) and older (age > 50 years) subgroups.

The ROC curve and the AUC comparing the ability to predict MetS of the BAI, the LAP, and the CMI are shown in Figure 2 and Figure 3, and in Table 3.

The BAI was found to be the lowest-performing index in comparison with the LAP and the CMI. In the general population, the ROC area was 0.50 (95% CI 0.47–0.52) while the optimal cut-off was found to be 43.55. With this cut-off, the sensitivity of the index was 30.31%, while the specificity was 74.48%, and the likelihood ratio was only 1.19. When the analysis was conducted on the male population, the ROC area was 0.51 (95% CI 0.42–0.59) while the optimal cut-off was 46.14. In this case, the sensitivity of the index decreased to 16.51%, while the specificity increased to 93.44%, and the likelihood ratio remained equal to 1.19. In the female population, the ROC area was 0.52 (95% CI 0.49–0.55) while the optimal cut-off was 56.79. With this cut-off, the sensitivity of the index was 16.89%, while the specificity was 89.70%, and the likelihood ratio increased to 1.64.

When the analysis was carried out on the younger population, the ROC area was 0.51 (95% CI 0.42–0.59) while the optimal cut-off was 56.173. In this case, the sensitivity of the index decreased to 13.41%, while the specificity increased to 91.20%, and the likelihood ratio was 1.52. In the older population, the ROC area was 0.50 (95% CI 0.46–0.54) while the optimal cut-off was 56.45. With this cut-off, the sensitivity of the index was 17.20%, while the specificity was 88.70%, and the likelihood ratio increased to 1.52.

The LAP and CMI indexes showed the best performance in discriminating the presence of MetS, both in the whole population and in the two genders considered separately. 

In particular, both the LAP and the CMI in the whole group showed a comparable ROC area of 0.82 (95% CI 0.80–0.54), while the optimal cut-offs were 91.05 for the LAP and 1.22 for the CMI. With these cut-offs, the LAP showed a sensitivity of 63.06%, a specificity of 86.55%, and a likelihood ratio of 4.69; while the CMI had a sensitivity of 67.59%, a specificity of 81.55%, and a likelihood ratio of 3.66. When the analysis was performed only in the male population, the LAP showed an ROC area of 0.81 (CI 95% 0.75–0.87), while the optimal cut-off was 101.5, showing a higher sensitivity of 70.81%, a lower specificity of 81.97%, and a lower likelihood ratio equal to 3.93. In the female population, the ROC area was 0.82 (95% CI 0.80–0.84), while the optimal cut-off was 87.39. With this cut-off, the sensitivity of the LAP was 64.27%, while the specificity was 85.45%, and the likelihood ratio of 3.93.

When the analysis was performed only in the younger population, the LAP showed an ROC area of 0.81 (CI 95% 0.78–0.84) while the optimal cut-off was 89.24, showing a sensitivity of 65.52%, a specificity of 83.37%, and a likelihood ratio equal to 3.93. In the older population, the ROC area was 0.82 (95% CI 0.79–0.84) while the optimal cut-off was 86.71. With this cut-off, the sensitivity of the LAP was 67.80%, while the specificity was 84.70%, and the likelihood ratio of 4.43.

As far as the CMI is concerned, in the male population, the ROC area was 0.81 (95% CI 0.76–0.87), while the optimal cut-off was 1.47, showing a sensitivity equal to 66.46%, a lower specificity equal to 85.25%, and a higher likelihood ratio equal to 4.50. In the female population, the ROC area was 0.81 (95% CI 0.79–0.83), while the optimal cut-off was 1.14. With this cut-off, the sensitivity of the CMI was 71.84%, while the specificity was 73.77%, and the likelihood ratio was 3.14.

When the analysis was performed in the younger population, the ROC area was 0.81 (95% CI 0.78–0.84), while the optimal cut-off was 1.25, showing a sensitivity equal to 71.83%, a specificity equal to 83.37%, and a likelihood ratio equal to 3.08. In the older population, the ROC area was 0.86 (95% CI 0.83–0.88), while the optimal cut-off was 1.12. With this cut-off, the sensitivity of the CMI index was 72.00%, while the specificity was 88.04%, and the likelihood ratio was 6.02.

The correlations between each index and some anthropometric and clinical characteristics in the whole study group and in the population divided into males and females are shown in Table 4.

Most variables were not significantly correlated with the indexes considered or showed a negligible correlation, regardless of whether the analysis was carried out on the entire population or the population divided according to sex. The BAI index in the entire population showed a weak correlation with WC and height and a moderate correlation with HC and BMI, while in both male and female subgroups, there was a weak correlation with WC, height, and BW and a moderate one with HC and BMI.

The LAP in the entire population showed a weak correlation with WC, glucose, and HDL-C concentrations and the presence of MetS, a moderate correlation with the CMI, and a strong correlation with triglyceride concentration. In the male population, there was a weak correlation with HDL-C concentration and the presence of MetS and a strong correlation with the triglyceride concentration and the CMI, while in the female population, there was a weak correlation with WC, glucose, and HDL-C concentrations and the presence of MetS, a moderate correlation with the CMI, and a strong correlation with the triglyceride concentration.

Lastly, the CMI in the whole study population, male and female populations, showed a weak correlation with HDL-C concentrations and the presence of MetS, and a strong correlation with the triglyceride concentration. Moreover, in the male population, the correlation of the CMI with the LAP index was strong, while it was moderate in both the general and the female populations.

## 4. Discussion

MetS is a systemic condition that combines a variety of metabolic alterations, and it is often present in patients with obesity [45]. Given the significant increase in the prevalence of both MetS and obesity [11,46] and the fact that MetS is a high-risk complication [8], its accurate and early identification could allow for individualized nutritional and/or pharmacological treatments which are crucial in preventing the occurrence of cardiovascular diseases and the mortality associated with the progression of those conditions [9,10,11,12].

For these reasons, the present study was aimed at assessing the accuracy of different indexes of metabolic and adiposity dysfunction (the BAI, the LAP, and the CMI) in identifying MetS [21,22,23,24,25,26,27,28,29] in a large cohort (no. 1912) of adult subjects with obesity. 

In our whole study group, the prevalence of MetS was 62.3%. As expected, almost all the parameters were significantly worse in the MetS+ subgroup than in the MetS− subgroup. In fact, the two subgroups were comparable in terms of hip circumference (HC), height, and BAI index values, while the MetS+ subgroup was significantly heavier (both in terms of body weight and BMI and older, with a greater WC and higher values of glycemia, cholesterolemia, triglyceridemia, and systolic and diastolic blood pressure. The LAP and the CMI values were also higher in the MetS+ subgroup than in the MetS− subgroup, while HDL cholesterol levels were significantly lower in the MetS+ subgroup.

In contrast to previous studies, in our study group, the prevalence of MetS was higher in males (72.5%) than in females (60.9%) [47,48,49,50,51]. However, even if it was less frequent, other studies reported cases of higher incidences of MetS in men with different ethnicities [51,52,53,54]. A possible explanation for these surprising results in our male population might be attributed both to the markedly different number of subjects recruited in the two subgroups (222 males vs. 1690 females) and to the relevant differences of BW (males: 126.9 kg vs. females: 107.6 kg) and WC (males: 131.5 cm vs. females: 120.1 females) between the two genders. In fact, even though, in other studies, patients diagnosed with MetS were mostly females, males often present more severe manifestations [55,56,57]. Another possible explanation could be that females with severe obesity are usually more willing to join a body weight reduction program (in-hospital) even in the absence of co-morbidities, such as diabetes, dyslipidemia, and hypertension. In contrast, males frequently decide to join a multidisciplinary in-hospital BWRP to lose weight only in the presence of other co-morbidities, thus having more probability to fulfill the criteria of having MetS.

When anthropometric characteristics and biochemical parameters are compared between the two sexes, males present a worse clinical picture than females. No significant gender-related differences were found in terms of BMI, glycemia, and cholesterolemia. By contrast, males showed significantly higher WCs, BWs, heights, triglycerides, systolic and diastolic blood pressures compared to females, while females were older, with greater HC and higher HDL-C values than males. As far as the three indexes are concerned, males showed higher LAP and CMI values than females, while females had higher BAI values than males. 

Of the three indexes considered, the LAP and the CMI performed better, being able to detect the presence of MetS in the whole study population and in male/female subgroups, other than in the younger/older subgroups.

In fact, unlike the BAI, which only takes into account anthropometric parameters, the LAP and the CMI (which also include fat distribution and the blood lipid profile) were significantly more sensitive and specific.

The BAI had the highest specificity value, thus being able to identify subjects without MetS more precisely, but had an extremely low sensitivity. These results are consistent with those already reported in other studies, where the BAI index was less accurate for the diagnosis of MetS [26,58,59,60].

The results obtained in terms of the likelihood ratio further confirm that the LAP and the CMI can be considered to be significantly better compared to the BAI, having a greater diagnostic accuracy. As far as the BAI is concerned, the likelihood ratio was the lowest and slightly higher than 1 in all cases (i.e., 1.19 in the whole study group and in females, and 1.64 in males), thus indicating that a subject who was positively identified by the BAI had only approximately one and a half times more probability of having MetS, compared to someone with a positive result but without MetS. On the contrary, the values found for the LAP and the CMI were much higher, both in the whole study population and in the population divided into males and females, suggesting that the two indexes had a much higher diagnostic accuracy. The likelihood ratios for the LAP were higher in the whole study population, and in females (4.69 and 3.93, respectively), while in males, the highest value (4.50) was recorded for the CMI. In these cases, a patient positively identified by the LAP and the CMI was approximately four times more likely to have MetS, compared to a subject identified as positive but without MetS.

To the best of our knowledge, this is the first study that has evaluated the performance of the BAI and both the LAP and the CMI in a cohort of both male and female adult individuals with obesity. It is not surprising that the LAP and the CMI were the best indexes identified since they demonstrated the highest sensitivity and specificity in recognizing patients with MetS in other study populations. For instance, the LAP was very effective in diagnosing MetS in women suffering from polycystic ovary syndrome who have a normal weight or who are overweight [61], in patients with HIV [24], as well as in the healthy adult population [32,62]. Similarly, the CMI was reported to be effective in discriminating between the presence or absence of MetS in adolescents and adult women with obesity [63,64]. Furthermore, the two indexes showed a good correlation between each other and regarding the presence of MetS, while the same correlations were not found with the BAI.

Our study presents some limitations. First, the sample size was very large but with a marked difference between the two genders (1690 females, 222 males), with this difference reflecting the real-life, usual proportion of patients admitted to our hospital. Ideally, the number of females should have been more similar to that of males. Another limitation is that the information about the comorbidities of each patient was not available, thus hampering the determination of both their frequency and the possible correlations with the indexes evaluated. Lastly, our results may not be representative of the entire population with obesity, since the study group was composed of Caucasian individuals with obesity, hospitalized for a multidisciplinary body weight reduction program. Therefore, it is not possible to generalize our conclusions to other ethnic groups and care settings.

On the contrary, the strength of this study lies in its large number of subjects with obesity, all recruited in the same third-level center for the multidisciplinary treatment of severe obesity. All patients were examined by a highly trained staff of physicians, and biochemical assessments were performed in the same central laboratory, thus abolishing the risk of inter-laboratories discrepancies.

## 5. Conclusions

In conclusion, the LAP and the CMI performed better than the BAI in detecting MetS in an adult population of both sexes with obesity, and may be considered to be useful and easy-to-be-determined tools to be introduced in clinical practice. In fact, the two indexes solely require the evaluation of WC and HC, along with the simultaneous evaluation of simple and routinely measurable biochemical parameters, such as blood triglycerides and C-HDL. 

Finally, it should be noted that, until fully validated, all the indexes analyzed in this study can only be considered as supporting tools for clinicians to perform an initial screening aimed at the early identification of the presence of MetS in populations with severe obesity who are at greater risk, without replacing the clinical assessment and the current diagnosis with the IDF criteria.

## Figures and Tables

**Table 1:** The main characteristics of the study population (total, patients with (MetS+) and without metabolic syndrome (MetS−).

	Total	MetS+	MetS−	*p*-Value
n.	1912	1191(62.3%)	721 (37.7%)	
Sex (F/M)	1690 (88.4%)/222 (11.6%)	1030 (86.5%)/161 (13.5%)	660 (91.5%)/61 (8.5%)	<0.001
Age (yrs)	50.7 ± 14.1	53.4 ± 12.6	46.2 ± 15.3	<0.0001
WC (cm)	121.4 ± 12.9	123.9 ± 12.7	117.4 ± 12.2	<0.0001
HC (cm)	131.9 ± 12.9	132.0 ± 13.5	131.7 ± 11.9	ns
BW (kg)	109.9 ± 18.8	111.0 ± 19.5	108.0 ± 17.4	<0.001
Height (cm)	159.1 ± 8.4	159.1 ± 8.4	159.2 ± 8.3	ns
BMI (kg/m^2^)	43.3 ± 6.2	43.8 ± 6.6	42.5 ± 5.4	<0.0001
SBP (mmHg)	128.6 ± 13.9	130.4 ± 13.6	125.5 ± 13.9	<0.0001
DBP (mmHg)	77.1 ± 7.8	77.6 ± 7.7	76.2 ± 7.9	<0.001
Glucose (mg/dL)	98.8 ± 32.2	108.0 ± 36.8	83.6 ± 11.9	<0.0001
T-C (mg/dL)	195.6 ± 37.2	198.1 ± 38.0	191.4 ± 35.5	<0.001
HDL-C (mg/dL)	49.6 ± 12.8	45.7 ± 11.4	56.1 ± 12.2	<0.0001
Triglycerides (mg/dL)	137.1 ± 65.7	159.5 ± 70.4	100.0 ± 32.3	<0.0001
BAI	48. 0 ± 7.8	48.1 ± 8.2	47.8 ± 7.8	ns
LAP	97.6 ± 52.0	116.4 ± 55.0	66.4 ± 24.9	<0.0001
CMI	1.44 ± 0.92	1.76 ± 1.0	0.93 ± 0.4	<0.0001

Abbreviations: WC, waist circumference; HC, hips circumference; BW, body weight; BMI, body mass index; SBP, systolic blood pressure; DBP, diastolic blood pressure; T-C, total cholesterol; HDL-C, HDL cholesterol; MetS, metabolic syndrome; BAI, the body adiposity index; LAP, the lipid accumulation product index; CMI, the cardiometabolic index.

**Table 2:** The characteristics of the study population, divided into groups of males and females.

	Males	Females	*p*-Value
n.	222 (11.6%)	1690 (88.4%)	
Age (yrs)	47.5 ± 14.7	51.1 ± 14.0	<0.001
WC (cm)	131.5 ± 12.6	120.1 ± 12.4	<0.0001
HC (cm)	130.1 ± 13.9	132.1 ± 12.7	<0.05
BW (kg)	126.9 ± 22.1	107.6 ± 17.1	<0.0001
Height (cm)	172.0 ± 7.9	157.4 ± 6.8	<0.0001
BMI (kg/m^2^)	42.8 ± 6.2	43.4 ± 6.2	ns
SBP (mmHg)	131.6 ± 15.1	128.2 ± 13.7	<0.001
DBP (mmHg)	78.2 ± 9.5	76.9 ± 7.6	<0.05
Glucose (mg/dL)	100.8 ± 35.7	98.5 ± 31.8	ns
T-C (mg/dL)	191.0 ± 35.0	196.2 ± 37.5	ns
HDL-C (mg/dL)	41.6 ± 10.0	50.7 ± 12.7	<0.0001
Triglycerides (mg/dL)	164.7 ± 87.0	133.4 ± 61.4	<0.0001
MetS (Y/N)	161 (72.5%)/61 (27.5%)	1030 (60.9%)/660 (39.1%)	<0.001
n. IDF criteria	3.2 ± 1.0	2.9 ± 1.2	<0.01
BAI	39.9 ± 6.9	49.1 ± 7.2	<0.0001
LAP	122.8 ± 67.0	94.3 ± 48.7	<0.0001
CMI	1.89 ± 1.3	1.38 ± 0.8	<0.0001

Abbreviations: WC, waist circumference; HC, hips circumference; BW, body weight; BMI, body mass index; SBP, systolic blood pressure; DBP, diastolic blood pressure; T-C, total cholesterol; HDL-C, HDL cholesterol; MetS, metabolic syndrome; BAI, the body adiposity index; LAP, the lipid accumulation product index; CMI, the cardiometabolic index.

**Table 3:** The ROC area, cut-off according to Youden Index, sensitivity, specificity, and likelihood ratio of the three indexes in the whole study population, in the male and female subgroups, and in the younger (age ≤ 50 years) and older subgroups (age > 50 years).

	ROC Area	Cut-Off	Sensitivity	Specificity	Likelihood Ratio
Population					
BAI	0.50 (0.47–0.52)	43.55	30.31%	74.48%	1.19
LAP	0.82 (0.80–0.84)	91.05	63.06%	86.55%	4.69
CMI	0.82 (0.80–0.84)	1.22	67.59%	81.55%	3.66
Males					
BAI	0.51 (0.42–0.59)	46.14	16. 51%	93.44%	1.19
LAP	0.81 (0.75–0.87)	101.5	70.81%	81.97%	3.93
CMI	0.81 (0.76–0.87)	1.47	66.46%	85.25%	4.50
Females					
BAI	0.52 (0.49–0.55)	56.79	16. 89%	89.70%	1.64
LAP	0.82 (0.80–0.84)	87.39	64.27%	85.45%	3.93
CMI	0.81 (0.79–0.83)	1.14	71.84%	73.77%	3.14
Younger (≤50 years)					
BAI	0.51 (0.42–0.59)	56.73	13. 41%	91.20%	1.52
LAP	0.81 (0.75–0.87)	89.24	65.52%	83.37%	3.93
CMI	0.81 (0.76–0.87)	1.25	71.83%	76.72%	4.50
Older (>50 years)					
BAI	0.50 (0.46–0.54)	56.45	17.20%	88.70%	1.52
LAP	0.82 (0.79–0.84)	86.71	67.80%	84.70%	4.43
CMI	0.86 (0.83–0.88)	1.12	72.00%	88.00%	6.02

**Table 4:** The correlations between each index and the anthropometric and clinical characteristics in the whole group and in the population divided into males and females.

		WC	HC	Height	BW	BMI	Glucose	T-C	HDL-C	Triglycerides	MetS	BAI	LAP	CMI
POPULATION														
BAI	*R* ^2^	0.116	0.580	0.320	0.054	0.499	0.004	<0.001	0.014	0.024	>0.001		<0.001	0.013
	*p*-value	<0.0001	<0.0001	<0.0001	<0.0001	<0.0001	<0.01	ns	<0.0001	<0.0001	ns		ns	<0.0001
LAP	*R* ^2^	0.193	0.011	0.016	0.066	0.045	0.103	0.079	0.132	0.850	0.217	>0.001		0.688
	*p*-value	<0.0001	<0.0001	<0.0001	<0.0001	<0.0001	<0.0001	<0.0001	<0.0001	<0.0001	<0.0001	ns		<0.0001
CMI	*R* ^2^	0.006	0.003	0.013	0.003	>0.001	0.053	0.028	0.355	0.823	0.193	0.013	0.688	
	*p*-value	<0.001	<0.05	<0.0001	<0.05	ns	<0.0001	<0.0001	<0.0001	<0.0001	<0.0001	<0.0001	<0.0001	
MALES														
BAI	*R* ^2^	0.358	0.645	0.202	0.139	0.548	0.017	0.004	0.005	0.009	0.004		0.009	>0.001
	*p*-value	<0.0001	<0.0001	<0.0001	<0.0001	<0.0001	ns	ns	ns	ns	ns		ns	ns
LAP	*R* ^2^	0.057	0.018	0.002	0.043	0.049	0.055	0.072	0.156	0.886	0.149	0.009		0.792
	*p*-value	<0.001	ns	ns	<0.01	<0.001	<0.001	<0.0001	<0.0001	<0.0001	<0.0001	ns		<0.0001
CMI	*R* ^2^	0.002	0.002	0.004	>0.001	>0.001	0.025	0.040	0.340	0.877	0.142	>0.001	0.792	
	*p*-value	ns	ns	ns	ns	ns	<0.05	<0.01	<0.0001	<0.0001	<0.0001	ns	<0.0001	
FEMALES														
BAI	*R* ^2^	0.244	0.648	0.215	0.174	0.567	0.004	0.001	0.002	0.012	0.003		>0.001	0.004
	*p*-value	<0.0001	<0.0001	<0.0001	<0.0001	<0.0001	0.0068	ns	ns	<0.0001	<0.05		<0.001	<0.05
LAP	*R* ^2^	0.201	0.013	0.001	0.047	0.050	0.116	0.091	0.112	0.837	0.230	0.007		0.651
	*p*-value	<0.0001	<0.0001	ns	<0.0001	<0.0001	<0.0001	<0.0001	<0.0001	<0.0001	<0.0001	<0.001		<0.0001
CMI	*R* ^2^	0.002	0.002	0.001	<0.001	<0.001	0.063	0.031	0.359	0.804	0.206	0.004	0.651	
	*p*-value	ns	ns	ns	ns	ns	<0.0001	<0.0001	<0.0001	<0.0001	<0.0001	<0.05	<0.0001	

## Data Availability

The datasets generated and analyzed in the present study are available upon a reasonable request to the corresponding author. Raw data will be uploaded to www.zenodo.org immediately after the acceptance of the manuscript.

## References

[1] Sideli L., Lo Coco G., Bonfanti R.C., Borsarini B., Fortunato L., Sechi C., Micali N. (2021). Effects of COVID-19 Lockdown on Eating Disorders and Obesity: A Systematic Review and Meta-Analysis. Eur. Eat. Disord. Rev. J. Eat. Disord. Assoc..

[2] Gammone M.A., D’Orazio N. (2021). COVID-19 and Obesity: Overlapping of Two Pandemics. Obes. Facts.

[3] De Lorenzo A., Romano L., Di Renzo L., Di Lorenzo N., Cenname G., Gualtieri P. (2020). Obesity: A Preventable, Treatable, but Relapsing Disease. Nutrition.

[4] Chooi Y.C., Ding C., Magkos F. (2019). The Epidemiology of Obesity. Metabolism.

[5] Blüher M. (2019). Obesity: Global Epidemiology and Pathogenesis. Nat. Rev. Endocrinol..

[6] Khanna D., Khanna S., Khanna P., Kahar P., Patel B.M. (2022). Obesity: A Chronic Low-Grade Inflammation and Its Markers. Cureus.

[7] Ellulu M.S., Patimah I., Khaza’ai H., Rahmat A., Abed Y. (2017). Obesity and Inflammation: The Linking Mechanism and the Complications. Arch. Med. Sci..

[8] Tarantino G., Caputi A. (2011). JNKs, Insulin Resistance and Inflammation: A Possible Link between NAFLD and Coronary Artery Disease. World J. Gastroenterol..

[9] Lee S.-Y., Chang H.-J., Sung J., Kim K.J., Shin S., Cho I.-J., Shim C.Y., Hong G.-R., Chung N. (2014). The Impact of Obesity on Subclinical Coronary Atherosclerosis According to the Risk of Cardiovascular Disease. Obesity.

[10] Lovren F., Teoh H., Verma S. (2015). Obesity and Atherosclerosis: Mechanistic Insights. Can. J. Cardiol..

[11] Bhupathiraju S.N., Hu F.B. (2016). Epidemiology of Obesity and Diabetes and Their Cardiovascular Complications. Circ. Res..

[12] Mathieu P., Pibarot P., Després J.-P. (2006). Metabolic Syndrome: The Danger Signal in Atherosclerosis. Vasc. Health Risk Manag..

[13] Zaha D.C., Vesa C., Uivarosan D., Bratu O., Fratila O., Tit D.M., Pantis C., Diaconu C.C., Bungau S. (2020). Influence of Inflammation and Adipocyte Biochemical Markers on the Components of Metabolic Syndrome. Exp. Ther. Med..

[14] Gheorghe G., Toth P.P., Bungau S., Behl T., Ilie M., Pantea Stoian A., Bratu O.G., Bacalbasa N., Rus M., Diaconu C.C. (2020). Cardiovascular Risk and Statin Therapy Considerations in Women. Diagn. Basel Switz..

[15] Engin A., Engin A.B., Engin A. (2017). The Definition and Prevalence of Obesity and Metabolic Syndrome. Obesity and Lipotoxicity.

[16] Pasquali R., Casanueva F., Haluzik M., van Hulsteijn L., Ledoux S., Monteiro M.P., Salvador J., Santini F., Toplak H., Dekkers O.M. (2020). European Society of Endocrinology Clinical Practice Guideline: Endocrine Work-up in Obesity. Eur. J. Endocrinol..

[17] Frankenfield D.C., Rowe W.A., Cooney R.N., Smith J.S., Becker D. (2001). Limits of Body Mass Index to Detect Obesity and Predict Body Composition. Nutrition.

[18] Gurunathan U., Myles P.S. (2016). Limitations of Body Mass Index as an Obesity Measure of Perioperative Risk. Br. J. Anaesth..

[19] Kok P., Seidell J.C., Meinders A.E. (2004). The value and limitations of the body mass index (BMI) in the assessment of the health risks of overweight and obesity. Ned. Tijdschr. Geneeskd..

[20] Bergman R.N., Stefanovski D., Buchanan T.A., Sumner A.E., Reynolds J.C., Sebring N.G., Xiang A.H., Watanabe R.M. (2011). A Better Index of Body Adiposity. Obesity.

[21] Kahn H.S. (2005). The “Lipid Accumulation Product” Performs Better than the Body Mass Index for Recognizing Cardiovascular Risk: A Population-Based Comparison. BMC Cardiovasc. Disord..

[22] Wakabayashi I., Daimon T. (2015). The “Cardiometabolic Index” as a New Marker Determined by Adiposity and Blood Lipids for Discrimination of Diabetes Mellitus. Clin. Chim. Acta Int. J. Clin. Chem..

[23] Tylutka A., Morawin B., Walas Ł., Michałek M., Gwara A., Zembron-Lacny A. (2023). Assessment of Metabolic Syndrome Predictors in Relation to Inflammation and Visceral Fat Tissue in Older Adults. Sci. Rep..

[24] Raposo M.A., Guimarães N.S., Tupinambás U. (2020). Lipid Accumulation Product Index to Predict Metabolic Syndrome in People Living with HIV. Clin. Med. Res..

[25] Kahn H.S. (2006). The Lipid Accumulation Product Is Better Than BMI for Identifying Diabetes. Diabetes Care.

[26] Siervo M., Prado C.M., Stephan B.C., Lara J., Muscariello E., Nasti G., Colantuoni A. (2014). Association of the Body Adiposity Index (BAI) with Metabolic Risk Factors in Young and Older Overweight and Obese Women. Eat. Weight Disord..

[27] Bullen A.L., Katz R., Kumar U., Gutierrez O.M., Sarnak M.J., Kramer H.J., Shlipak M.G., Ix J.H., Judd S.E., Cushman M. (2022). Lipid Accumulation Product, Visceral Adiposity Index and Risk of Chronic Kidney Disease. BMC Nephrol..

[28] Ayundini G., Astrella C., Tahapary D., Soewondo P. (2019). A Systematic Review on the Association between Lipid Accumulation Product Index and Type 2 Diabetes Mellitus. J. ASEAN Fed. Endocr. Soc..

[29] Ahn N., Baumeister S.E., Amann U., Rathmann W., Peters A., Huth C., Thorand B., Meisinger C. (2019). Visceral Adiposity Index (VAI), Lipid Accumulation Product (LAP), and Product of Triglycerides and Glucose (TyG) to Discriminate Prediabetes and Diabetes. Sci. Rep..

[30] Zhao S., Ren Z., Yu S., Chi C., Tang J., Maimaitiaili R., Teliewubai J., Li J., Xu Y., Zhang Y. (2021). Association Between Lipid Accumulation Product and Target Organ Damage in Elderly Population: The Northern Shanghai Study. Clin. Interv. Aging.

[31] Golabi S., Ajloo S., Maghsoudi F., Adelipour M., Naghashpour M. (2021). Associations between Traditional and Non-Traditional Anthropometric Indices and Cardiometabolic Risk Factors among Inpatients with Type 2 Diabetes Mellitus: A Cross-Sectional Study. J. Int. Med. Res..

[32] Taverna M.J., Martínez-Larrad M.T., Frechtel G.D., Serrano-Ríos M. (2011). Lipid Accumulation Product: A Powerful Marker of Metabolic Syndrome in Healthy Population. Eur. J. Endocrinol..

[33] Shi Y., Hu L., Li M., Zhou W., Wang T., Zhu L., Bao H., Li P., Cheng X. (2021). Relationship Between the Lipid Accumulation Product Index and Arterial Stiffness in the Chinese Population With Hypertension: A Report From the China H-Type Hypertension Registry Study. Front. Cardiovasc. Med..

[34] Almeda-Valdés P., Cuevas-Ramos D., Aguilar-Salinas C.A. (2009). Metabolic Syndrome and Non-Alcoholic Fatty Liver Disease. Ann. Hepatol..

[35] Anoop S.S., Dasgupta R., Rebekah G., Jose A., Inbakumari M.P., Finney G., Thomas N. (2021). Lipid Accumulation Product (LAP) as a Potential Index to Predict Risk of Insulin Resistance in Young, Non-Obese Asian Indian Males from Southern India: Observations from Hyperinsulinemic-Euglycemic Clamp Studies. BMJ Open Diabetes Res. Care.

[36] Dursun M., Besiroglu H., Otunctemur A., Ozbek E. (2016). Association between Cardiometabolic Index and Erectile Dysfunction: A New Index for Predicting Cardiovascular Disease. Kaohsiung J. Med. Sci..

[37] Zou J., Xiong H., Zhang H., Hu C., Lu S., Zou Y. (2022). Association between the Cardiometabolic Index and Non-Alcoholic Fatty Liver Disease: Insights from a General Population. BMC Gastroenterol..

[38] Wang H.-Y., Shi W.-R., Yi X., Wang S.-Z., Luan S.-Y., Sun Y.-X. (2018). Value of Reduced Glomerular Filtration Rate Assessment with Cardiometabolic Index: Insights from a Population-Based Chinese Cohort. BMC Nephrol..

[39] Wang H., Chen Y., Sun G., Jia P., Qian H., Sun Y. (2018). Validity of Cardiometabolic Index, Lipid Accumulation Product, and Body Adiposity Index in Predicting the Risk of Hypertension in Chinese Population. Postgrad. Med..

[40] Duan S., Yang D., Xia H., Ren Z., Chen J., Yao S. (2022). Cardiometabolic Index: A New Predictor for Metabolic Associated Fatty Liver Disease in Chinese Adults. Front. Endocrinol..

[41] Lohman T.G., Roche A.F., Martorell R. (1991). Anthropometric Standardization Reference Manual: Abridged Edition.

[42] Alberti K.G.M.M., Eckel R.H., Grundy S.M., Zimmet P.Z., Cleeman J.I., Donato K.A., Fruchart J.-C., James W.P.T., Loria C.M., Smith S.C. (2009). Harmonizing the Metabolic Syndrome: A Joint Interim Statement of the International Diabetes Federation Task Force on Epidemiology and Prevention; National Heart, Lung, and Blood Institute; American Heart Association; World Heart Federation; International Atherosclerosis Society; and International Association for the Study of Obesity. Circulation.

[43] Youden W.J. (1950). Index for Rating Diagnostic Tests. Cancer.

[44] Schober P., Boer C., Schwarte L.A. (2018). Correlation Coefficients: Appropriate Use and Interpretation. Anesth. Analg..

[45] Belladelli F., Montorsi F., Martini A. (2022). Metabolic Syndrome, Obesity and Cancer Risk. Curr. Opin. Urol..

[46] Vishram J.K.K., Borglykke A., Andreasen A.H., Jeppesen J., Ibsen H., Jørgensen T., Palmieri L., Giampaoli S., Donfrancesco C., Kee F. (2014). Impact of Age and Gender on the Prevalence and Prognostic Importance of the Metabolic Syndrome and Its Components in Europeans. The MORGAM Prospective Cohort Project. PLoS ONE.

[47] Radetti G., Fanolla A., Grugni G., Lupi F., Tamini S., Cicolini S., Sartorio A. (2021). The Role of Different Indexes of Adiposity and Body Composition for the Identification of Metabolic Syndrome in Women with Obesity. J. Clin. Med..

[48] Pucci G., Alcidi R., Tap L., Battista F., Mattace-Raso F., Schillaci G. (2017). Sex- and Gender-Related Prevalence, Cardiovascular Risk and Therapeutic Approach in Metabolic Syndrome: A Review of the Literature. Pharmacol. Res..

[49] Krieger N. (2003). Genders, Sexes, and Health: What Are the Connections—And Why Does It Matter?. Int. J. Epidemiol..

[50] Regitz-Zagrosek V., Oertelt-Prigione S., Prescott E., Franconi F., Gerdts E., Foryst-Ludwig A., Maas A.H.E.M., Kautzky-Willer A., EUGenMed, Cardiovascular Clinical Study Group (2016). Gender in Cardiovascular Diseases: Impact on Clinical Manifestations, Management, and Outcomes. Eur. Heart J..

[51] Beigh S.H., Jain S. (2012). Prevalence of Metabolic Syndrome and Gender Differences. Bioinformation.

[52] Ahonen T., Saltevo J., Laakso M., Kautiainen H., Kumpusalo E., Vanhala M. (2009). Gender Differences Relating to Metabolic Syndrome and Proinflammation in Finnish Subjects with Elevated Blood Pressure. Mediat. Inflamm..

[53] Njelekela M.A., Mpembeni R., Muhihi A., Mligiliche N.L., Spiegelman D., Hertzmark E., Liu E., Finkelstein J.L., Fawzi W.W., Willett W.C. (2009). Gender-related differences in the prevalence of cardiovascular disease risk factors and their correlates in urban Tanzania. BMC Cardiovasc. Disord..

[54] Fezeu L., Balkau B., Kengne A.P., Sobngwi E., Mbanya J.C. (2007). Metabolic syndrome in a sub-Saharan African setting: Central obesity may be the key determinant. Atherosclerosis.

[55] Henstridge D.C., Abildgaard J., Lindegaard B., Febbraio M.A. (2019). Metabolic Control and Sex: A Focus on Inflammatory-Linked Mediators. Br. J. Pharmacol..

[56] Li W., Qiu X., Ma H., Geng Q. (2022). Incidence and Long-Term Specific Mortality Trends of Metabolic Syndrome in the United States. Front. Endocrinol..

[57] Alemany M. (2024). The Metabolic Syndrome, a Human Disease. Int. J. Mol. Sci..

[58] Elisha B., Rabasa-Lhoret R., Messier V., Abdulnour J., Karelis A.D. (2013). Relationship between the Body Adiposity Index and Cardiometabolic Risk Factors in Obese Postmenopausal Women. Eur. J. Nutr..

[59] Schulze M.B., Thorand B., Fritsche A., Häring H.U., Schick F., Zierer A., Rathmann W., Kröger J., Peters A., Boeing H. (2012). Body Adiposity Index, Body Fat Content and Incidence of Type 2 Diabetes. Diabetologia.

[60] Shin K.-A., Hong S.B., Shin K.S. (2017). Body Adiposity Index and Metabolic Syndrome Risk Factors in Korean Adults: A Comparison with Body Mass Index and Other Parameters. Biomed. Sci. Lett..

[61] Han W., Zhang M., Wang H., Yang Y., Wang L. (2023). Lipid Accumulation Product Is an Effective Predictor of Metabolic Syndrome in Non-Obese Women with Polycystic Ovary Syndrome. Front. Endocrinol..

[62] Tellechea M.L., Aranguren F., Martínez-Larrad M.T., Serrano-Ríos M., Taverna M.J., Frechtel G.D. (2009). Ability of Lipid Accumulation Product to Identify Metabolic Syndrome in Healthy Men from Buenos Aires. Diabetes Care.

[63] Radetti G., Grugni G., Lupi F., Fanolla A., Caroli D., Bondesan A., Sartorio A. (2022). High Tg/HDL-Cholesterol Ratio Highlights a Higher Risk of Metabolic Syndrome in Children and Adolescents with Severe Obesity. J. Clin. Med..

[64] Lazzer S., D’Alleva M., Isola M., De Martino M., Caroli D., Bondesan A., Marra A., Sartorio A. (2023). Cardiometabolic Index (CMI) and Visceral Adiposity Index (VAI) Highlight a Higher Risk of Metabolic Syndrome in Women with Severe Obesity. J. Clin. Med..

